# The Effects of Sampling Lateralization on Bilateral Inferior Petrosal Sinus Sampling for Pediatric Cushing’s Disease—A Single Endocrinology Centre Experience and Review of the Literature

**DOI:** 10.3389/fendo.2021.650967

**Published:** 2021-04-19

**Authors:** Elzbieta Moszczyńska, Elzbieta Marczak, Mieczysław Szalecki, Krzysztof Kądziołka, Marcin Roszkowski, Patrycja Zagata-Lesnicka

**Affiliations:** ^1^ Department of Endocrinology and Diabetes, The Children’s Memorial Health Institute, Warsaw, Poland; ^2^ Collegium Medicum, The University of Jan Kochanowski, Kielce, Poland; ^3^ Department of Neurosurgery, The Children’s Memorial Health Institute, Warsaw, Poland; ^4^ Department of Clinical Research, Allmedica Clinical Research Center, Nowy Targ, Poland

**Keywords:** Cushing’s disease, pituitary adenoma, BIPSS, hypercortisolemia, lateralization, adenoma

## Abstract

**Background:**

This study aims to analyze the diagnostic accuracy of bilateral inferior petrosal sinus sampling (BIPSS), the gold standard test for the differential diagnosis of ACTH-dependent Cushing’s syndrome (CS) in a group of pediatric patients with Cushing’s disease (CD).

**Methods:**

This is a retrospective analysis which include 12 patients with hypercortisolemia and inconclusive pituitary MRI, who underwent bilateral inferior petrosal sinus sampling (BIPSS) and transsphenoidal surgery (TSS) from 2004 to 2020 in the Children’s Memorial Health Institute (CMHI) Warsaw, Poland. Pituitary origin of ACTH secretion was considered if baseline central to peripheral (C/P) ACTH level ratio was ≥ 2 or C/P ratio was ≥ 3 after human corticotropin-releasing hormone (hCRH) stimulation. The diagnosis was histologically confirmed in almost all cases after TSS.

**Results:**

The diagnostic accuracy of BIPSS reached 75% at baseline and 83.3% after CRH stimulation. The compatibility of localization of a microadenoma by BIPSS with the surgical location was 66.7%.

**Conclusions:**

Owing to its high diagnostic effectiveness, BIPSS remains the best test to differentiate CD from EAS. The indications for the procedure should be carefully considered, because EAS in the pediatric population, unlike in adults, is extremely rare. Moreover BIPSS has only limited value for indicating tumor localization.

## Introduction

Cushing’s disease (CD) is caused by an ACTH-secreting pituitary adenoma and is the most common cause of Cushing’s syndrome in children over 7 years of age (1–3). An accurate diagnostic pathway is highly desirable, as untreated CD may lead to various morbidities as well as higher mortality. Biochemical diagnosis of CS is based on the measurement of cortisol circadian rhythmicity, urine free cortisol (UFC), and low dose dexamethasone suppression test (LDDST). Baseline adrenocorticotropic hormone (ACTH) was measured to differentiate between ACTH-dependent and ACTH-independent CS. Corticotropin-releasing hormone (CRH) stimulation, and high dose dexamethasone suppression test (HDDST) can be used as second line test to reinforce the diagnosis of CD. To detect the localization of adenoma contrast-enhanced magnetic resonance imaging (MRI) is performed. Since 90% of the pituitary adenomas in CD are microadenomas (smaller than 1 cm in greatest diameter) and 70% having a diameter of ≤5mm, MRI often fails to detect the lesion (1). Nowadays, bilateral inferior petrosal sinus sampling (BIPSS) with CRH stimulation, is the most reliable method since it offers the highest approachable sensitivity and specificity in confirming pituitary ACTH production exceeding 95% (4, 5). BIPSS has also been used for the purpose of lateralization of a pituitary adenoma to one or the other side of the pituitary gland, thus guiding the surgeon during transsphenoidal surgery (TSS). Sensitivity for lateralization by BIPSS has been reported to be ranging from 54% up to 100% (4, 6–11).

The first-line treatment for ACTH-secreting adenomas is transsphenoidal surgery, and the aim of the surgery is a selective removal of intra-pituitary adenomas with a preservation of normal pituitary tissue, which is especially important in the pediatric population (1). In case of an unsuccessful surgery or relapse resurgery, radiotherapy, pharmacological treatment, and very rarely bilateral adrenalectomy are considered.

We describe a series of 12 children treated in one specialist center, all of whom underwent the BIPSS procedure. The purpose of this study was to evaluate the diagnostic effectiveness of BIPSS before and after hCRH stimulation as a tool for the localization of small corticotroph adenomas in pediatric CD. The relevant literature concerning the management of BIPSS in pediatric CD was reviewed to demonstrate the role of this procedure in investigating patients with suspected CD.

## Materials and Methods

### Patients

Throughout the years 1993-2020, 33 children were diagnosed with CD in the Children’s Memorial Health Institute (CMHI), Warsaw, Poland. This retrospective longitudinal study covered the period ranging from 2004 to 2020. We evaluated 12 children who were admitted to the CMHI for clinical evaluation and underwent BIPSS due to the presence of Cushing’s syndrome. The study group consisted of 6 male and 6 female patients, the average age of first hypercortisolemia symptoms was 8.5 years (age range 6.3 – 10 yrs). The average duration of symptoms before treatment was 2.5 years (range 0.8 - 8.2 yrs).

During the 27-year observation period, only 2 patients were diagnosed with Cushing’s syndrome due to ectopic ACTH secretion. They were not qualified for BIPSS because CT/MRI scans showed the presence of extrapituitary tumors produced ACTH (thymus tumor and tumor of the appendix).

Ethical approval has been obtained from our local hospital Ethics Committee.

### Endocrine Evaluation

The diagnosis of Cushing’s disease was based on the following biochemical criteria: hypercortisolemia, confirmed by the loss of serum cortisol circadian rhythm with an elevated midnight serum cortisol (≥1.8 μg/dl), 4x increase above normal 24h measurement of UFC, and failure of serum cortisol to suppress to less than 1.8 ug/dl on low dose dexamethasone suppression test (LDDST). To differentiate between ACTH-dependent and ACTH-independent causes basal serum ACTH was measured. Corticotropin dependence was defined by concentration ≥10 pg/mL. The diagnosis of Cushing’s disease was supported by suppression of serum cortisol to more than 50% of basal values in a high dose dexamethasone suppression test (HDDST), and exaggerated serum cortisol and ACTH increase of more than 20% and 35% respectively, as compared with baseline values, during CRH stimulation test (12).

Cortisol samples were measured with one-step immunoassay (Architect, Abbot), whereas ACTH was assayed with a radioimmunoassay (ELSA-ACTH, Cisbio, Codolet, France). UFC was assayed by HPLC (high-performance liquid chromatography).

### Pituitary Imaging

After pituitary hormone analysis, it is essential to investigate the accurate location of the suspected adenoma. In our study group, we obtained a dynamic gadolinium-enhanced MRI of the pituitary gland using a 1.5 T scanner (Siemens).

### BIPSS Protocol

Due to unsuccessful detection of adenoma localization in MRI, all mentioned patients with documented ACTH-dependent CS underwent BIPSS with hCRH stimulation. Hypercortisolism was additionally confirmed prior to approaching this invasive technique.

The procedures were performed by experienced interventional radiologists or neurosurgeons, 2 sinus samplings took place in London. Patients’ parents were fully informed about the procedure and signed a written consent. The investigation protocol was conducted as described by Oldfield et al. (1, 4).

Patients were examined under general anesthesia. 4F catheters were guided into an orifice of each of the right and left inferior petrosal sinuses *via* a femoral vein approach, under radiographic guidance. The position of catheters was confirmed radiologically, by retrograde venography, which is based on the injection of a contrast dye. Systemic anticoagulation with heparin was routinely used.

Blood samples were obtained simultaneously from both IPSs and from a peripheral vein *via* the sheath in one of the femoral veins before (-1 and 0 min) and after (+2-3, +5, and +10 min) iv administration of 1μg/kg human CRH (hCRH), max.100μg (Ferring Pharmaceuticals). All collected samples were immediately collected in chilled test tubes containing sodium ethylenediaminetetraacetic acid (EDTA), placed on ice, and delivered for immunoradiometric (RIA) ACTH assay.

### BIPSS Interpretation

The test was considered diagnostic of a pituitary origin of ACTH secretion if baseline central to peripheral (C/P) ACTH level ratio was ≥ 2 or post-CRH C/P ratio was ≥ 3 (1, 4).

The position of the adenoma was indicated if the intersinus ratio was greater than 1.4 either before or after CRH injection. An IPS ratio smaller than 1.4 was considered as evidence for the lack of lateralization and was used as evidence of a middle lesion (13).

### Surgical and HistopathologicalData Analysis

After BIPSS all of the patients underwent transsphenoidal selective adenectomy. Procedures were performed by two experienced neurosurgeons, under a surgical microscope approach. During the surgery, the lateralization of adenoma was defined and suspected tumor tissue was prepared for histopathological analysis. Some specific immunohistochemistry data including ACTH, Ki-67 index, and somatostatin receptor (SSTR) expression were measured.

### Therapeutic Outcome

Postsurgical remission was defined as normal 24-h urine free cortisol, normal midnight serum cortisol, a normal LDDST or continued need for hydrocortisone, assessed periodically (14).

Hypercortisolism after TSS, lack of a diurnal rhythm with the increased nocturnal serum cortisol value >1.8μg/dl was defined as persistent disease (15).

Recurrence was defined as the return of clinical manifestations with biochemical evidence of hypercortisolism (16).

### Statistical Analysis

Continuous variables are presented as the median or the mean ± standard deviation. Qualitative variables include features related to the location of the adenoma, the treatment outcome, and histopathological evaluation.

## Results

The study group consisted of 12 patients (6 male and 6 female), all of whom met the criteria of the study and had BIPSS performed due to inconclusive biochemical and MRI results (**Table 1**).

**Table 1:** BIPSS results in pediatric population with Cushing disease.

Patient	Pre-oCRH C/P ratio	Post-oCRH stimulation C/P ratio	Tumor location			Treatment outcome	Histopathological confirmation^a^
			MRI	BIPSS basal/CRH stimulation	Surgery		
1	21.5	63.2	normal	R	R	no relapse	microadenoma
2	9.3	23.3	normal	L	C	no relapse	microadenoma
3	0.8	1.1	heterogenous	lateralization (-)	C	no relapse	microadenoma
4	1.5	1.4	normal	lateralization (-)	L	no relapse	microadenoma
5	3.7	19.5	3mm (L)	L	L	no relapse	microadenoma
6	14.9	27.0	heterogenous	R	R	no relapse	microadenoma
7	8.3	22.4	4mm (R)	R	R	2 x surgery RTX bilateral adrenalectomy	1.microadenoma 2.microadenoma
8	1.3	5.6	normal	R	C	2 x surgery	1.microadenoma 2.microadenoma
9	94.5	750.2	normal	R	L	no relapse	corticotropin cell hyperplasia, IHC ACTH (+)
10	43.4	115	heterogenous	R	R	no relapse	pituitary
11	15.8	20.3	heterogenous	R	R	no relapse	microadenoma, IHC ACTH (-)
12	30.7	303.4	normal	L	L	2 x surgery	1.microadenoma, IHC ACTH (+) 2.microadenoma

R, right; L, left; IHC, immunohistochemistry.

a IHC staining for ACTH was performed since 2016.

The median age at the onset of the symptoms was 8.5 years (range 6.3-10), patients were diagnosed at 11.6 years (range 7.2–17.0), the median duration of disease manifestations before diagnosis was 2.5 years (range 0.8–8.2). All subjects had typical features of a patient with Cushing’s syndrome. The main presenting features were growth retardation in association with weight gain, acne, plethora, hirsutism, easy bruising, mental and behavioral problems, hypertension, glucose intolerance. Clinical features and long-term outcomes have already been described in a study on 29 pediatric patients with CS diagnosed and treated in CMHI between 1993-2018 (17). **Table 2** shows the laboratory assessment of the patients.

**Table 2:** Baseline characteristics and biochemical evaluation of 12 pediatric patients with Cushing’s disease.

No.	Sex	Age at symptoms onset	Age at the time of diagnosis	Duration of disease^a^ (years)	Midnight cortisol (μg/dl)	ACTH (pg/ml)N 10-60	24h UFC (μg)	Increase after CRH stimulation		Morning serum cortisol (μg/dl)		
								ACTH (%)	Cortisol (%)	0’	LDDST (48h)	HDDST (48h)
1	M	15.7	16.7	5	13.3	66	480.8 (<100)	43.8	47	N.A.	N.A.	N.A.
2	F	9.4	10.6	1.2	12.9	51	849.8 (4-34)	437.2	107	26.8	4.8	13.1
3	M	7	15.2	8.2	23	128	322.3 (13-120)	226	96	21	2.2	1.7
4	F	10	12	2.0	17.3	44.3	605 (8.6-52.4)	259.2	139	20.3	11.4	2.2
5	F	8	14.3	6.3	12.6	38	220.7 (8.6-52.4)	29.5	50	20	5.3	2.6
6	M	10	17	7.0	13	43	379.6 (13-120)	0 ^b^	0 ^b^	18	1.6	1
7	M	9	11.6	2.6	49.1	45	626 (8-65)	35.1	12	43.9	44.3	22.8
8	F	6	8.5	2.5	17.8	69	260.4 (2.1-23)	41.2	50	24.2	2	1.1
9	M	8	10.4	2.4	7.9	118	201.4 (8-65)	334.9	295	11.2	0.7	0.5
10	M	10	11.7	1.7	11.8	80.2	839.2 (8-65)	35	20	25.9	19.7	1.9
11	F	6.3	7.2	0.8	10.4	30.7	944 (2.1-23)	143.3	56	30.2	23.8	20
12	F	7.0	11	4.0	12.9	55.2	284 (4-34)	214.1	205	15.2	10.4	3.9

a time interval from the onset of initial symptoms to TSS.

b in one patient, neither ACTH nor cortisol increased during CRH test.

### Laboratory Evaluation

CD was diagnosed in all patients with hypercortisolemia, confirmed by the loss of serum cortisol circadian rhythm - midnight serum cortisol levels > 1.8 μg/dl, with values ranging from 7.9 μg/dl up to 49.9 μg/dl, median 12.9 μg/dl.

UFC was measured at least twice, based on the 24-hour urine collection, exceeding the normal at least 4 times.

Serum ACTH in all the cases was either within the normal range or elevated, the median level of basal plasma ACTH was 75.8 pg/ml, which confirmed ACTH dependence.

The majority (9/11; 81.8%) of subjects failed to suppress the secretion of serum cortisol < 1.8 μg/dl during LDDST. However, 2 patients suppressed serum cortisol to 1.6 μg/dl and 0.7 μg/dl in LDDST.

In the HDDST test, 9/11 (81.8%) patients met the cut-off criteria of over 50% basal cortisol level suppression. Among the remaining ones, one of the patients reached 48% cortisol suppression and the another one 34%.

CRH test supported the diagnosis of CD in 9/12 (75%) cases as the subjects exhibited an increase of ACTH level of at least 35%, as well as an increase in serum cortisol of at least 20%. In one case none of the rises were sufficient to confirm the diagnosis of CD. In two cases either ACTH or cortisol rise criteria were not fulfilled.

A detailed hormonal assessment of each patient is shown in **Table 2**.

### Pituitary Imaging

MRI scanning was performed in all the subjects, though 10 (83.3%) patients had no visible adenoma, in 2 cases (16.7%) diagnosis of adenoma ≤ 4 mm was made, and in 4 cases (33.3%) heterogeneously enhancing on T1 weighted (T1W) imaging was obtained in sagittal and coronal planes. Due to inconclusive MRI reports, all the patients of the study group underwent BIPSS.

### Additional Imaging

While qualifying for BIPSS, all the patients had either MRI or CT imaging of the chest and abdomen and underwent SSTR scintigraphy of the whole body. Only patients with negative results of these tests were qualified for BIPSS.

### BIPSS

**Figure 1:**
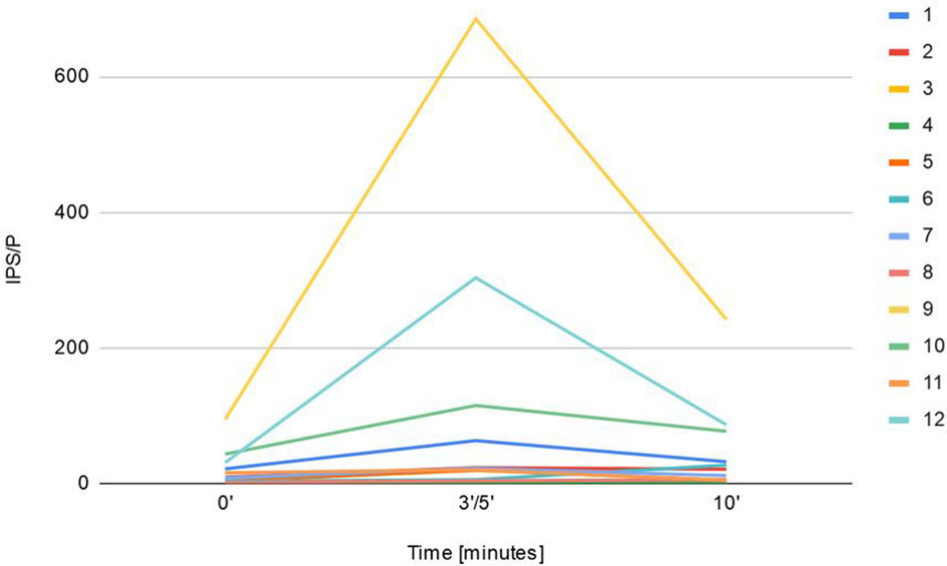
Presentation of maximal IPS/P ACTH gradient at baseline and after CRH administration (3'-5' and 10') during BIPSS procedure in 12 children.

Sampling with CRH stimulation was performed successfully in all 12 cases. Baseline ACTH secretion measured in peripheral veins, dominant and nondominant petrosal sinus reached 60 pg/mL (± 45.8), 391.5 pg/mL (± 2619.2), 96.5 pg/mL (± 201.6) respectively. The maximum basal ACTH level at the petrosal sinus was 9200 pg/mL. Peak ACTH after CRH stimulation at the periphery, dominant and nondominant sinus equaled 148.5 pg/mL (± 129.2), 1323.5 pg/mL (± 44587.3), 327 pg/mL (± 5353.8) respectively. Central ACTH secretion was confirmed in 75% (9/12) unstimulated procedures, where IPS/P ratio was ≥ 2 and in 83.3% (10/12) stimulated samplings with the ratio ≥ 3. The studied group had the highest IPS/P ACTH ratio before and after CRH stimulation that reached 94.5 and 750.2 respectively, and concerned the above-mentioned patient. The maximum ACTH concerned mentioned patient and reached 156 800 pg/ml 5 minutes after CRH stimulation. IPS/P ratio of two patients did not meet the criteria for CS diagnosis, though histopathology from TSS confirmed the presence of ACTH-secreting tumors (**Figure 1**).

ACTH lateralization at dominant IPS was present before stimulation in 8/12 cases (66.7%) and after CRH stimulation was successful in 10/12 (83.3%) of the undertaken samplings.

**Figure 2:**
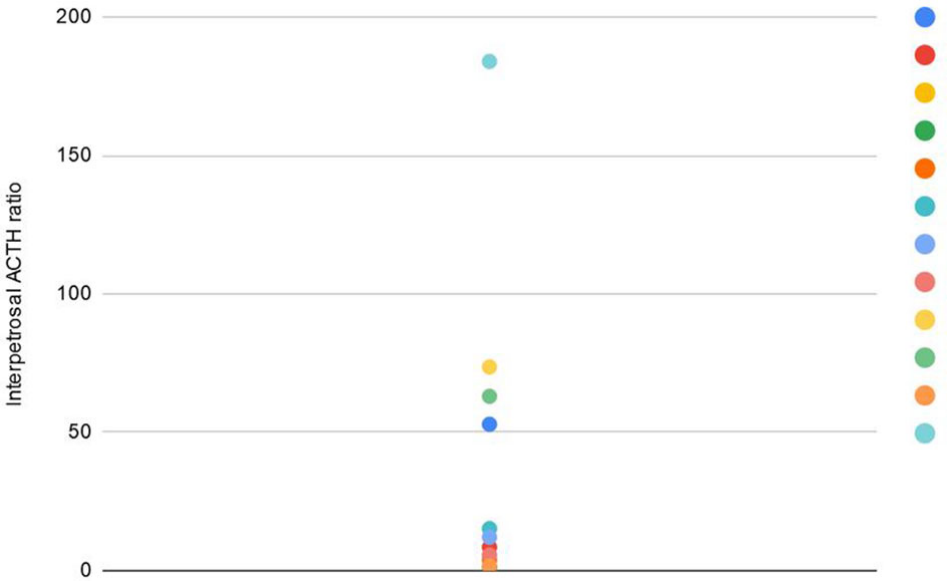
Maximal interpetrosal ACTH ratio during BIPSS in the studied group.

BIPSS lateralization concordance with the surgical findings was rated 58.3% (7/12). In two cases (16.7%) the ratio of 1.4 was not reached, which complied with the TSS finding of a central tumor. Lesion location of the other three patients (3/12, 25%) was not compatible with the BIPSS lateralization results (**Figure 2**).

Detailed baseline characteristics of study participants are demonstrated in **Table 3**. There were no severe complications associated with the procedure, in several cases catheterization led to hematomas of the groin at the site of venipuncture.

**Table 3:** Literature summary of BIPSS outcome in Cushing’s disease in children.

Study	Study cohort	Mean age; age range (years)	Study duration	Baseline C/P ratio > 2	Post-CRH/desmopressin stimulation C/P ratio >3	Compatibility of localization of a microadenoma by BIPSS with surgical location
**Lienhardt et al.** (8)	11	12.3 ± 3.9; 10.6-18.8	1986-2000	N.A.	91% (ratio >2)	91%^d^
**Joshi et al.** (18)	25 (19 BIPSS)	13.4 6.6-17.8	1984-2003	N.A.	100%	73.7%, 94%^d^
**Batista et al.** (19)	94	13 ± 3.2; 5.3 - 18.7	1982-2004	90%	97%	60.3% (baseline), 58%(CRH stimulation)
**Shah et al.** (20)	48 (14 BIPSS)	14.9 ± 2.5; 9-19	1988-2008	89%^a^	N.A.	N.A.
**Storr et al.** (21)	41 (33 BIPSS)	12.3 ± 3.5; 5.7-17.8	1960-2001	75.9%	86.2%	75.8%, 81.8%^d^
**Lonser et al.** (22)	140	N.A.; 5.8-20.8	1982-2010	N.A.	99.3^b^	81.8%
**Yordanova et al.** (23)	21 (19 BIPSS)	12.1; 5.7-17.8	1986-2010	N.A.	N.A.^b^	74%
**Güemes et al.** (24)	30	8.9; 0.2–15.5	1983-2013	100%	100%	N.A.
**Chen et al.** (25)	16 (17 BIPSS)	15.5 ± 2.9; 9.8-18.7	2006-2017	64.7%	83.3% ^c^	50% (baseline), 42.9%^c^
**Cavalcante et al.** (26)	19	14; 9-19	1993-2017	84.2%	94.4%^c^	60%^c^
**Authors’ series**	31 (12 BIPSS)	11.6; 7.2-17	2003-2020	75%	83.3%	58.3%, 66.7% ^d^

a baseline ratio criteria ≥ 2.

b peak ratio criteria ≥3.

c desmopressin stimulation.

d total prediction rate (lateralization and no lateralization in case of central adenoma localization).

### Surgical Treatment

All the patients were operated on *via* a transsphenoidal approach as a first-line treatment. The adenoma was visible during surgery in all the patients. In 10 patients, the adenoma was confirmed histologically. In another 2 patients corticotropin cell hyperplasia and normal pituitary were found (both in remission). In 3 patients (25%) histopathological findings confirmed ACTH (+) in IHC in intraoperatively excised tumors. The surgical remission rate was 83.3% (10/12) patients. Two patients (2/12, 16.7%) had persistent hypercortisolemia and required a second surgery, one additionally underwent pituitary irradiation. One patient died after the second operation due to a stroke in the perioperative period.

### Further Treatment and Follow-Up

Median follow-up was 10.1 years (0.7-24.5 yrs). Among hormonal complications, long term ACTH deficiency was present in 83.3% of patients (10/12) who required further replacement treatment. Half of the studied group had permanent diabetes insipidus (DI), another three patients (25%) had transient postoperative DI. Additional postoperative complications encountered TSH deficiency (83.3%, 10/12), as well as gonadotropin deficiency (75%, 9/12). GH deficiency was recognized in 11 among 12 patients (91.6%), and led to rhGH treatment in 8 cases (66.7%).

Three patients (3/12, 25%) were qualified to the second TSS: in two cases remission was not reached after the first TSS: the first patient was operated on for the second time within 5 months, having a complete pituitary removal. As the second surgery did not lead to a remission, the patient was qualified for radiotherapy, which led to remission. The recurrence took place after 3.5 yrs, the patient eventually underwent a successful bilateral adrenalectomy. The second patient had a resurgery after almost 3 months following initial TSS, which led to a remission. The third patient achieved remission after the initial surgery, though she had a recurrence during the follow-up. The second TSS, a complete hypophysectomy was performed 2.5 yrs after the first surgery. Ischemic stroke marked the postoperative course, leading to the death of the patient.

### Genetics

Tumor tissue of eight patients from the analyzed group was screened for somatic USP 8 mutation, but it was not detected in any case. Additionally, none of the analyzed patients had symptoms characteristic for the genetic syndromes that include pituitary adenomas.

## Discussion

The study presents the results of BIPSS in a cohort of 12 children managed over years in a tertiary pediatric endocrinology service. The differential diagnosis of CS and especially ACTH-dependent CS is a challenging issue for clinical management. Neither biochemical nor imaging tests currently used exhibit a satisfactory sensitivity. Sensitivity and specificity for HDDST are estimated at 65-100% and 60-100% respectively and for CRH test 70-93% and 95-100% respectively (5). 90% of tumors in CD are microadenomas, in 50% cases undetectable in gadolinium MRI (1). BIPSS is believed to exhibit the highest sensitivity among the available procedures distinguishing CD and EAS, namely 95-99% in adult patients (4). Until now, indications for BIPSS in children have not been accurately specified. On the contrary, in the adult guidelines, the role of BIPSS in the diagnostic algorithm is well established: discordant tests, negative MRI image or microadenomas smaller than 6 mm (27). BIPSS should be recommended in patients with ACTH-dependent CS whose clinical, biochemical, or radiological studies are discordant or equivocal, according to the consensus on the management of Cushing’s syndrome (16). Other authors, including Oldfield E.H and Newell-Price J., suggest that BIPSS should be performed in most patients with ACTH-dependent CS, because the results of biochemical tests with CD and EAS may overlap (some neuroendocrine tumors may express glucocorticoids and/or CRH and/or vasopressin receptors). Additionally, the incidence of pituitary incidentaloma at autopsy ranges from 1.4% up to 27% of the general population and between 3.7% and 37% on imaging, usually <5 mm (5, 12, 16, 18, 28). We use guidelines for the adult population. However, it needs to be highlighted that ectopic ACTH secretion in adults accounts for 10-20% cases of ACTH-dependent Cushing’s syndrome while being extremely rare within the pediatric age (17, 29). Out of 33 pediatric ACTH-dependent Cushing’s syndrome cases investigated at CMHI between 1993 and 2020, only 2 had ectopic ACTH syndrome (neuroendocrine tumor of the thymus and appendix), which corresponds to 6.5%, none of whom had BIPSS performed (30). EAS represented 11% cases of ACTH -dependent CS in the study by Güemes et al. on a cohort of 30 children managed over 30 years in a tertiary pediatric endocrinology service (24). The French study identified 10 cases of EAS in children and adolescents under 20 years during a 23-year period from 11 adult and 7 pediatric endocrine departments of university hospitals (31). The majority of EAS described in children are case reports. More et al. compiled 47 of them, including those from the French study (31). Out of 306 cases of pediatric and adult Cushing’s syndrome investigated at St. Bartholomew’s Hospital during 28 years, 32 had ectopic ACTH syndrome, of whom only 1 was of pediatric age (12).

Since 1995, we have been performing BIPSS with hCRH stimulation in children with ACTH-dependent Cushing’s syndrome, in cases with equivocal results of hormonal tests and/or MRI negative for the presence of adenoma or focal lesions ≤4mm. The mean age of the patients who underwent BIPSS was 11.6 years, which corresponds to the data from the literature, the youngest patient was 7.2 years old.

All analyzed patients had normal or above serum level of ACTH and a UFC at least 4x above normal. Also, all described patients had midnight serum cortisol above 7.9 μg/dl, which may lead to recognizing the cut-off point of hypercortisolemia proposed by Papanicolaou et al. of above 7.5 μg/dl with sensitivity 97% (32). 2 patients in this group, with histologically proven Cushing’s disease, suppressed cortisol to <1.8 μg/dl in LDDST. Newell-Price and Storr also described such cases in 2% and 8% patients respectively (21, 33). False-negative LDDST results may be due to intermittency or periodic hypercortisolism. Another reason may be mild hypercortisolism with still preserved sensitive of ACTH secreting cells to glucocorticoid negative feedback (34).

Even though cortisol suppression in HDDST of at least 50% was reported as being typical for Cushing’s disease, it was not observed in 2 cases from the study group, amounting to only 48% and 34% (35). According to Nieman et al., HDDST fails to suppress urinary 17-hydroxycorticosteroid secretion by 50% in about 20% of patients with CD (36). In the study by Storr et al., 7% pediatric patients with CD did not suppress the serum cortisol <50% baseline during HDDST (21). Some authors undermine the validity of using HDDST in CD diagnostics (21). Batista et al. suggest a cut-off point of >20% for the cortisol suppression in CD, with 97.5% sensitivity (37). Based on a CRH stimulation test, 3 patients from the study group did not meet the CD recognition criteria introduced by Nieman et al. (38). In the case of one patient, a rise in neither ACTH nor cortisol was observed, despite confirmed microadenoma in histopathological examination. In another case 50% cortisol rise was reached with an ACTH increase of only 29.5%, in the final case, the ACTH increase criteria were fulfilled (35.1%) while the cortisol rise was not high enough, as it only reached 12% (**Table 3**).

Diagnostic errors due to the CRH stimulation test occur with a frequency of 8-15% in CD (21, 36).

Only in 2 patients, MRI showed a focal lesion ≤4 mm, in the remaining cases no pituitary adenoma was found, despite repeated imaging studies.

Given the biochemical discrepancy and/or the absence of a tumor on the MRI, we requested the BIPSS in all 12 patients being the main goal to confirm pituitary ACTH secretion and exclude ectopic ACTH production, and to define lateralization of the pituitary adenoma to one or the other side of the pituitary gland. All the interventions took place without severe complications.

An IPS/P baseline ratio ≥2 and after a CRH peak ≥3 was present in 75% (9/12) and 83.3% (10/12) CD patients respectively, which indicated the pituitary gland as the source of excessive ACTH secretion. 2 patients who did not meet the mentioned ratio criteria, had adenoma removed, confirmed by histopathological examination, leading to effective operations. None of the patients has been diagnosed with ectopic ACTH syndrome. In literature false negative results have been reported to be approximately 10%, and may be related to operational failure or abnormal venous drainage from the inferior petrosal sinus. For BIPSS, the success rate is closely related to the operator’s technique and experience. Results of a previous study suggest that prolactin for correction improves the success rate of catheterization (10).

Data on BIPSS outcomes in the pediatric population are limited (literature review - **Table 3**).

First significant series on 50 pediatrician patients with ACTH-dependent Cushing’s syndrome who had BIPSS was published in 1994 (1). Since then, other articles have been reported that accounted for over 500 patients, with the great majority having CD. The literature data presented in **Table 3** show that baseline ACTH IPS/P ratio ≥2 ranged from 64.7 to 100%, and after CRH/desmopressin stimulation ≥ 3 from 50 to 100%, but it has almost always been 100% in larger series (8, 19–23, 25, 26, 29, 31).

The second objective of the BIPSS is to help with surgical tumor localization by predicting the results of intersinus gradient descent. It is very important in the pediatric population because selective resection of pituitary adenoma with preservation of normal pituitary tissue is especially important for this group of patients.

In our study group, ACTH sampling correctly predicted the site of adenoma in 66.7% cases including central localization.

Literature data show that BIPSS lateralization complies with the result during surgery by 54-100%. For stimulation during BIPSS ovine CRH (oCRH) is used, rather than human CRH (hCRH), only a few authors mention performing the procedure with desmopressin (25, 26).

Based on the literature, it is known that oCRH is a stronger stimulant than hCRH, so it is difficult to compare individual results of the studies (39). Experience with desmopressin in BIPSS is limited (40, 41). As shown in the study, administration of CRH in the BIPSS test does not significantly improve the diagnostic accuracy of the test, though a correct diagnosis even in very few cases is most valuable. Some authors emphasize the limitation of BIPSS in determining the lateralization of ACTH secreting adenoma in pediatric CD (only 54-56%), stating that the integration of MRI findings and BIPSS cannot predict the location of the tumor more frequently than MRI alone (19, 42).

Similarly, in the report by Crock et al., BIPSS was shown not to be reliable for ACTH adenoma localization, as the procedure was performed in 13 patients, 46% of whom had false adenoma lateralization (43).

Similarly, Batista et al. showed that BIPSS is a poor prediction of the site of the adenoma (58%) (19). Chen et al. estimate the accordant rate of lateralization at only 50% (25).

On the contrary, other authors suggest that BIPSS is the best method available for the intrapituitary localization of microadenoma causing CD (8, 15, 18). In a study by Joshi et al. lateralization rate was 73.7%, but after taking into account the central position it reached 94%. The limitation of the mentioned study (18) was a small group of participants (19 patients).

BIPSS gives a 91% prediction of correct tumor localization in a small group of patients, as described by Lienhardt et al., but as was suggested by the authors in a larger series lateralization rate is likely to decrease (8).

In the studied group, the only complication of BIPSS was hematoma of the groin at the site of venipuncture. Single patient of the analyzed group died as a result of the stroke in the perioperative period after the second TSS performed due to recurrence tumor. The impact of the BIPSS conducted 2 years prior to death cannot be established.

## Conclusion

According to literature BIPSS is the best test to differentiate CD from EAS with very high specificity and sensitivity, better than other biochemical tests. Diagnostic effectiveness of BIPSS has also been confirmed in our study. However, we are aware that the sensitivity rate of the method in the pituitary location of the tumor showed only limited value.

Since BIPSS is an invasive procedure it is mandatory that BIPSS is carried out in a selected group of patients, bearing in mind that EAS occurs in the child population very rarely. BIPSS should be performed by experienced operators trained in this type of procedure to avoid not only potential serious complications, but also improper procedures and data misinterpretation.

The diagnosis of ACTH-dependent Cushing’s syndrome must be ascertained before BIPSS is performed and hypercortisolism before examination should be confirmed, to avoid testing during the inactive cycled-out phase of CD/EAS.

## Glossary

ACTH, adrenocorticotropic hormone; BIPSS, bilateral inferior petrosal sinus sampling; CD, Cushing’s disease; CMHI, Children’s Memorial Health Institute; CRH, corticotropin-releasing hormone; CS, Cushing’s syndrome; DI, diabetes insipidus; hCRH, human corticotropin-releasing hormone; EAS, ectopic ACTH syndrome; EDTA, ethylenediaminetetraacetic acid; ELISA, enzyme-linked immunosorbent assay; HDDST, high dose dexamethasone suppression test; HPLC, high-performance liquid chromatography; IHC, immunohistochemistry; IPS, inferior petrosal sinus; LDDST, low dose dexamethasone suppression test; MRI, magnetic resonance imaging; oCRH, ovine corticotropin-releasing hormone; RIA, radioimmunological assays; SSTR – somatostatin receptor; TSS, transsphenoidal surgery; UFC, urine free cortisol

## Data Availability Statement

The raw data supporting the conclusions of this article will be made available by the authors, without undue reservation.

## Ethics Statement

Ethical review and approval was not required for the study on human participants in accordance with the local legislation and institutional requirements. Written informed consent to participate in this study was provided by the participants’ legal guardian/next of kin.

## Author Contributions

EMo contributed to conception and design of the study. Resources were gathered by EMo, MS, KK, and MR. EMo and EMa organized the database. Statistical analysis was performed by EMa and PZ-L. EMo wrote the first draft of the manuscript. EMa wrote sections of the manuscript. All authors contributed to the article and approved the submitted version. EMo supervised preparation of the content.

## Conflict of Interest

The authors declare that the research was conducted in the absence of any commercial or financial relationships that could be construed as a potential conflict of interest.
